# EHMTI-0144. Physical and mental health status of people with medication-overuse headache across socioeconomic strata: results of a population-based study

**DOI:** 10.1186/1129-2377-15-S1-B39

**Published:** 2014-09-18

**Authors:** M Westergaard, C Glümer, E Holme Hansen, RH Jensen

**Affiliations:** 1 Department of Neurology, Danish Headache Center, Glostrup, Denmark; 2 Glostrup Hospital, Research Center for Prevention and Health, Glostrup, Denmark; 3 Department of Pharmacy, Faculty of Health and Medical Sciences, Copenhagen, Denmark

## Introduction

Medication-overuse headache (MOH) is more prevalent among those with low socioeconomic position (SEP), but it is not known how SEP influences the physical and mental health status of people with MOH.

## Aim

In this cross-sectional study, we compared health status scores of people with MOH to the mean for the population, stratified according to SEP indicators: educational attainment, work status, and income.

## Methods

129,150 individuals aged >16 years were invited to the 2010 Danish National Health Survey. Data on SEP indicators were retrieved from national registers. Respondents with headache >15 days/month over three months were classified as having chronic headache (CH). Those with CH and concurrent over-the-counter analgesic intake of >15 days/month were classified as having medication-overuse headache (MOH). Physical and mental health composite scores (SF-12) were summarized per headache group, stratified by SEP, and compared to the sample mean. All analyses were adjusted for stratified sampling and non-response.

## Results

The response rate was 53.1%. The prevalences of CH (3.3%) and MOH (1.7%) were inversely related to SEP. Compared to the general population, health status scores were significantly lower among people with CH, particularly those with MOH, regardless of education and income. Scores were markedly lower among those with MOH who were unemployed, early pensioners, or were receiving social/sickness benefits.

## Conclusion

CH and MOH are more prevalent among those with low SEP but is associated with physical and mental ill-health across all socioeconomic strata. Preventing and treating MOH would substantially reduce the individual and societal burden of CH.

No conflict of interest.

